# Editorial: Strategies in the drug discovery and development for leishmaniasis: immunomodulators, natural products, synthetic compounds, and drug repositioning

**DOI:** 10.3389/fcimb.2024.1384244

**Published:** 2024-03-13

**Authors:** Taís Fontoura de Almeida, Edézio Ferreira Cunha-Junior, João Luiz Mendes Wanderley, Juliana da Silva Pacheco, Lucia Helena Pinto-da-Silva, Valter Viana Andrade-Neto, Suzana Passos Chaves

**Affiliations:** ^1^ Laboratory of Physiopathology, Biosciences Applied to Health Department, Institute of Medical Sciences, Multidisciplinary Center of Federal University of Rio de Janeiro, Macaé, Brazil; ^2^ Laboratory of Immunoparasitology, Institute of Pharmaceutical Sciences, Multidisciplinary Center of Federal University of Rio de Janeiro, Macaé, Brazil; ^3^ Laboratory of Immunoparasitology, Biosciences Applied to Health Department, Institute of Medical Sciences, Multidisciplinary Center of Federal University of Rio de Janeiro, Macaé, Brazil; ^4^ Drug Discovery Unit, Department of Biological Chemistry and Drug Discovery, School of Life Sciences, University of Dundee, Dundee, United Kingdom; ^5^ Institute of Veterinary, Department of Veterinary Microbiology and Immunology, Seropédica, RJ, Brazil; ^6^ St Michael Hospital, Li ka Shing Knowledge Institute, Toronto, ON, Canada

**Keywords:** treatment, chemotherapy, immunomodulation, nanoparticles, repositioning drugs, flavonoid, protozoa, *Leishmania*

Leishmaniasis is a group of tropical diseases that affect millions of people worldwide. These diseases are transmitted by vectors and caused by protozoa of the genus *Leishmania*. The WHO reported that in 2020, Leishmaniasis was present in 98 countries or territories, and it posed a severe public health issue in four eco-epidemiological regions: Americas, East Africa, North Africa, West and Southeast Asia. That year, there were reports of 208,357 cases of Cutaneous Leishmaniasis (CL) and 12,838 cases of Visceral Leishmaniasis (VL) (Ruiz-Postigo et al., 2021). For the last 70 years, the available chemotherapy has been constituted by first-line (pentavalent antimonials) and second-line drugs (amphotericin B, pentamidine, paramomycin, and miltefosine). Existing drugs generally have disadvantages related to therapeutic approaches, such as route of administration, the need for trained professionals for administration, toxicity, high cost. Considering the number of pathogenic species concerning therapeutic options, this arsenal still needs to be improved (Burza et al., 2018). The lack of a vaccine or effective chemotherapy has stimulated many studies involving the research of new molecules or drug repositioning and new strategies and technologies in treating Leishmaniasis with potential microbicidal and immunomodulators.

The approach to developing chemotherapeutic strategies for Leishmaniasis is a comprehensive understanding of the disease’s immunology aspects, aiming to identify new drug targets and target the immune response against the protozoa. Magalhães et al. conducted a clinical trial treating patients with meglumine antimoniate plus N-acetylcysteine (SbV+NAC) and with meglumine antimoniate (SbV). The SbV+NAC group exhibited elevated soluble CD40-ligand levels, negatively correlated with interleukin-10 (IL-10). NAC reduced IL-10 production in monocytes and altered T-cell responses in stimulated peripheral blood mononuclear cells. Although the TNF-α/IL-10 ratio increased, suggesting an immunomodulatory role for NAC, it did not directly affect parasite load in infected macrophages. Despite immunological alterations, the pilot trial did not reveal significant clinical outcome differences between SbV and SbV+NAC groups. The study supports NAC’s safety and immunomodulatory potential in VL but recommends further research on its clinical efficacy, optimal dosages, and mechanisms in *Leishmania* infection therapy. Conde et al.,discussed diverse aspects of the immune response in Leishmaniasis, emphasizing factors like antigenicity, host immunity, sandfly saliva, and parasite load. The work highlights the role of B cells and humoral immunity, suggesting that the balance between immunoglobulin classes indicates different disease stages. It underscores the complex role of B cell subpopulations and antibody subtypes since different B cell subpopulations may have detrimental or beneficial roles for disease progression and immune response evasion. This review presents challenges for vaccine development due to the inaccurate information regarding the dynamics of B cells during the infection, emphasizing the need for future research on both cellular and humoral adaptive responses. Noronha et al. investigated the role of leukotrienes, particularly cysteinyl leukotrienes LTC4 and LTD4, in resisting *L. amazonensis* infection. Previous data indicated LTB4 production’s role in infection control. However, this study revealed that treatment with cysteinyl leukotrienes resulted in fewer amastigotes in macrophages and reduced cutaneous lesion progression in mice. The paper explores the link between ATP-induced cysteinyl leukotrienes (Cys-LTs) production triggered by the P2X_7_ receptor, showing that *L. amazonensis* infection downregulates Cys-LTs production and P2X_7_-deficient macrophages exhibit reduced Cys-LTs production. These findings highlight the importance of Cys-LTs as mediators for infection control and potential targets for CL treatment, suggesting the need for further mechanistic studies to understand the P2X_7_-LT axis’s leishmanicidal effects and its association with macrophage activation, cytokine production, and immune response.

It is also necessary to search for new alternatives for actual treatment and several natural products have been demonstrated to have antileishmanial activities. Quercetin is a polyphenolic flavonoid well known for its antioxidant activity in radical scavenging and other biological effects (Gervazoni et al., 2020). Dos Santos et al., demonstrated the therapeutic potential of oral quercetin in hamsters infected with *L. braziliensis*. Quercetin has both direct antileishmanial activity and the potential to modulate macrophages’ activity since there was a reduction in the parasite load both in the lesion and the draining lymph node. In contrast, treatment with oral administration of apigenin controls infection but does not compromise the overall health of the infected mice. So, apigenin, another flavonoid, satisfies all eligibility’s criteria of a new drug to treat VL and supports further studies to determine the ideal therapeutic and optimal drug dose regimen (Emiliano et al.). Rebouças-Silva et al. highlight the effects of lignans isomers, yangambin and epi-yangambin, as leishmanicidal and immunomodulatory molecules for *in vitro* infection with *L. amazonensis* and *L. braziliensis*. Either lignan attenuated the inflammatory profile of infected cells, but epi-yangambin was more potent in reducing the intracellular viability of *L. amazonensis* than yangambin. These leishmanicidal effects are related to a direct impact on intracellular parasites and macrophage activation. Pinheiro et al. showed that Farnesol, derived from farnesyl pyrophosphate in the sterols biosynthetic pathway, interferes with the proliferation of *L. amazonensis* promastigotes, inhibiting the cell cycle without causing DNA fragmentation or loss of mitochondrial functionality.

Another important approach is drug repurposing, which involves identifying new therapeutic uses for existing drugs developed for a different medical condition. Several drugs for treating Leishmaniasis have been repositioned (Andrade-Neto et al., 2018). Barroso et al. evaluated the effectiveness of meglumine antimoniate and liposomal amphotericin B in Brazil’s *L. braziliensis* endemic area. The results demonstrated that meglumine antimoniate displayed a higher cure rate but presented a greater rate of adverse events than liposomal amphotericin B. van Henten et al. explored the treatment of CL using cryotherapy and miltefosine in Ochollo (Ethiopia). One hundred forty-seven patients were treated with these strategies, revealing that better clinical outcomes and lesion remission were shown for miltefosine. However, there was poor adherence to miltefosine treatment, which explained the lower cure rates observed in this study. These findings reinforce the importance of decentralizing therapy and the need for extensive efforts to sensitize and instruct rural communities on treatment adherence. In addition to drug repositioning, a combination of drugs can be used in leishmaniasis treatment. Borges et al. investigated the anti-*leishmania* activity of triclabendazole, used to treat fascioliasis, combined with amphotericin B against *L. amazonensis*. The treatment with triclabendazole demonstrated morphological alteration and synergic effect with amphotericin B against intracellular amastigotes. Meira et al. showed the activity of the synthetic lapachol derivative 3-phenyl-lawsone (3-PL) *in vitro* and its therapeutic potential in experimentally infected hamsters through subcutaneous and tattooing route compared with meglumine antimoniate. 3-PL exerts a dose-dependent effect on *L. braziliensis* and in previous studies against *L. amazonensis*. The use of tattooing for drug delivery proved efficacious in reducing parasite load for 3-PL and Glucantime, delivered by tattooing for the first time. Jara et al. studied a panel of *L. braziliensis* strains highly susceptible to potassium antimonyl tartrate (PAT). Exposed promastigotes to lethal PAT pressure and compared several cellular and molecular parameters distinguishing temporary quiescence or survival drug tolerance from resistance against anti-leishmanial drugs. Resistance against anti-*Leishmania* drugs has been studied for years, giving meaningful insights into the long-term adaptations of these parasites to drugs through genetic modifications. However, microorganisms can also survive lethal drug exposure by entering temporary quiescence, a phenomenon called drug tolerance, which is rather unexplored in *Leishmania*.

These promising studies related to the discovery of new molecules or drug repositioning, new strategies, and alternative protocols in the treatment of CL and VL were welcome and a step forward to other efforts to treat this complex disease.

## Author contributions

TdA: Writing – review & editing. EC-J: Writing – review & editing. JW: Writing – original draft. JP: Writing – original draft. LP: Writing – original draft. VA-N: Writing – original draft. SC: Writing – review & editing.
